# Motivational Climate, Anxiety and Physical Self-Concept in Trainee Physical Education Teachers—An Explanatory Model Regarding Physical Activity Practice Time

**DOI:** 10.3390/ijerph191912812

**Published:** 2022-10-06

**Authors:** Eduardo Melguizo-Ibáñez, Félix Zurita-Ortega, José Luis Ubago-Jiménez, Pilar Puertas-Molero, Gabriel González-Valero

**Affiliations:** Department of Didactics of Musical, Plastic and Corporal Expression, Faculty of Education Sciences, University of Granada, 18071 Granada, Spain

**Keywords:** disruptive states, active lifestyle, body image, education science students

## Abstract

There is an increase in sedentary lifestyles among young people. However, the development of a certain motivational climate can play a key role in the prevention of such lifestyles. Taking into account the aforementioned, the present research aims to establish the relationship between the motivational climate towards sport, anxiety and physical self-concept and to identify and clarify the existing relationships between anxiety, motivational climate and physical self-concept, by breaking down this objective into (a) developing an explanatory model of the motivational climate towards sport and its relationship with anxiety and physical self-concept and (b) contrasting the structural model by means of a multi-group analysis, according to the time spent doing physical activity per week. For this purpose, a cross-sectional descriptive and comparative study was carried out with a total of 568 university students (M = 25.09; SD = 6.22). A sociodemographic questionnaire, the Spanish version of the Perceived Motivation Climate Questionnaire in Sport, the Self-Concept Form-5 and the Beck Anxiety Inventory were used for data collection. The results show that more time spent doing physical activity brings benefits in terms of physical self-concept and homework climate, helping to reduce anxiety levels. As conclusions, it is observed that a longer time spent doing physical activity brings benefits in the channelling of disruptive states and improvements in physical self-concept.

## 1. Introduction

In the adult population, there is an increase in sedentary lifestyles in different western societies due to a decrease in the time spent doing physical activity [1]. This concept can be defined as a bodily movement of any kind produced by muscular contraction and which results in a substantial increase in energy expenditure in a person [2]. These low levels have a direct impact on trainee physical education teachers’ physical and mental health, as it has been shown that regular physical exercise provides numerous health benefits [3], including the prevention of type 2 diabetes [4], reduction of blood pressure [5], prevention of heart attacks and cerebral thrombosis [5]. The WHO states that the adult population should engage in 150–300 min of moderate-intensity aerobic physical activity per week to maintain a healthy lifestyle, with an improvement in health occurring when 300 min per week is exceeded [6]. The organisation also points out that such recommendations help to improve mental health, by preventing and reducing the onset of disruptive states such as depression and anxiety [7].

One of the most studied factors in sport psychology is motivation, which can be defined as a mechanism that controls the intensity and direction of efforts due to its great potential to explain different human behaviours [8,9]. In the theory of achievement goals [10], it is found that the term ‘perception’ originated from the motivational climate, and is defined as the set of indicators that different subjects perceive of their environment through which success or failure in the performance of a certain activity is defined [11], so the creation of a certain motivational climate will depend on the motivations from which the subject orients the performance of a certain task. Specifically, within the physical sports environment, when the practice of physical activity is oriented towards mastery, values such as fun or personal satisfaction (task climate) become important, while when extrinsic values are emphasised, competition is encouraged (ego climate) [12], thus generating an increase in the levels of frustration and anxiety when the proposed objectives are not achieved [13]. Likewise, it has been observed that when physical sports practice is guided by an intrinsic motivation, these behaviours are repeated in later stages of human development [14]. Acquiring healthy behaviours can help to improve peoples’ physical and psychological health.

Anxiety can be defined as a state of worry that is difficult for the subject to control and is associated with symptoms, such as irritability, difficulty concentrating and muscle tension [15]. In this case, the university environment causes an increase in anxiety levels due to the academic load to which students are subjected [16]. Research by Kayani et al. [16] states that intrinsically motivated physical exercise helps to reduce levels of this disruptive state. Furthermore, Rumbold et al. [17] state that when a sport begins to be performed professionally, there is an increase in anxiety, as participants focus on competitiveness, obviating the enjoyment and fun of the sport.

It has been shown that in the youth population, physical aptitude is another element that promotes anxiety levels [18]. Specifically, within the field of psychology, one of the most studied factors related to physical apprehension is physical self-concept [17]. Physical self-concept is defined as peoples’ perception of their own physical appearance [19]. The study by Fraguela-Vale et al. [20] states that people who regularly exercise have a better body self-concept than those who do not. Furthermore, body dissatisfaction can lead to high levels of anxiety, as research by Lee [21] states that a person not accepting themselves physically as they are, is positively related to a higher level of anxiety, which may be due to social pressure put on appearance and a personal dissatisfaction related to body image for not meeting the standards of beauty, as established by society [22]. In this case, the adolescent and university populations are the most affected by the pursuit of these beauty standards, which can lead to unhealthy behaviours in order to achieve them [22].

Therefore, the present study originates from the following question: Does the motivational climate towards which the practice of physical activity is oriented, influence the development of anxiety and physical self-concept? Does a longer time spent practicing physical activity help to improve the associations between motivational climate, anxiety and physical self-concept? Focusing attention on the research hypotheses, it is established that:

**H.1.**  *Participants who engage in more than 300 min of physical activity per week will show a greater adherence to the motivational climate task and higher physical self-concept scores.*

**H.2.**  *Participants who engage in more than 300 min of physical activity per week will show lower levels of anxiety than those who engage in less physical activity per week*.

**H.3.**  *Subjects who practice less than 150 min per week will show worse climate task scores and worse physical self-concept scores than those who practice more than 150 min per week*.

**H.4.**  *Subjects who practice less than 150 min per week will show higher levels of anxiety than those who practice more than 300 min per week of physical activity*.

Finally, the current research evidences the general objectives of establishing the relationship between motivational climate, anxiety and physical self-concept and identifying and clarifying the existing relationships between anxiety, motivational climate and physical self-concept, broken down into (a) developing an explanatory model of motivational climate and its relationship with anxiety and physical self-concept and (b) contrasting the structural model by means of a multi-group analysis according to the time spent doing physical activity per week.

## 2. Materials and Methods

### 2.1. Design of the Study and Participants

A quantitative, non-experimental (ex post facto), comparative, cross-sectional study was carried out. No manipulation of any of the variables was carried out. The study sample consisted of 568 trainee physical education teachers (M = 25.09; SD = 6.22) belonging to the Faculty of Education Sciences of the University of Granada. In terms of gender, 140 were male while 428 were female. In this case, the inclusion criterion was that the participants belonged to a university degree in primary education and that they had taken a physical education course. In this case, the failure to comply with the above was an exclusion criterion. In accordance with the procedure followed to carry out the research, first of all, a bibliographical review was carried out in order to gain a deeper understanding of the problems addressed. Then, from the Department of Didactics of Musical, Plastic and Corporal Expression of the University of Granada, a Google Form was created with the instruments described above, the research objectives and the informed consent of the participants. The virtual medium was used for data collection, mainly due to the health crisis caused by COVID-19. To avoid random responses, two questionnaires were duplicated, but fourteen had to be deleted as they were incorrectly completed.

Finally, convenience sampling was used to select the participants, who had to be related to the degrees taught in the aforementioned faculty, which was assumed to be an exclusion criterion. In terms of sampling error, for a maximum error for a confidence level of 95%, an error of 3.17% was achieved.

### 2.2. Instruments and Variables

**Sociodemographic questionnaire:** This is a self-drafted sheet where socio-demographic and physical sporting aspects are collected by means of a self-recording. In this case, variables such as the sex and age of the participants were recorded. For the variables aimed at the physical sports domain, questions about compliance with the physical activity recommended by the WHO [23] of less than 150 min, between 150 and 300 min and more than 300 min per week [12], were used to determine whether they were physically active or not [24,25].

**Beck Anxiety Inventory:** Developed by Beck et al. [26], for this study, we used the Spanish version developed by Sanz and Navarro [27]. This questionnaire is composed of a total of 21 items, which are measured on a four-level Likert-type scale (0 = not at all and 3 = very much). For this research, Cronbach’s alpha obtained a score of 0.956.

**Perceived Motivational Climate in Sport Questionnaire (PMCSQ-2):** Developed by Newton et al. [28]. However, the version adapted to Spanish by González-Cutre et al. [29] was used. This questionnaire is made up of a total of 33 items that are assessed using a five-level Likert scale (1 = strongly disagree and 5 = strongly agree). The instrument also assesses motivation through two motivational climates. The first is the task climate which is made up of three dimensions: effort improvement (EI), cooperative learning (CL) and important role (IR). The second motivational climate is the ego climate which is made up of three further sub-dimensions: unequal recognition (UR), punishment for mistakes (PM) and member rivalry (MR). Regarding the reliability analysis, Cronbach’s alpha obtained a score of α = 0.934, while the ego climate obtained a score of α = 0.962.

**Self-Concept Questionnaire Form 5:** Developed by García and Musitu [30]. It consists of a total of 30 items, assessed through a Likert scale with values ranging from 1 (Never) to 5 (Always). For the present research, only the items that make up the physical self-concept (5, 10, 15, 20, 25, 30) were used. The reliability obtained for this questionnaire was α = 0.885.

### 2.3. Ethical Approval

The present study followed, at all times, the principles established by the Helsinki Declaration of 1975 and was approved and supervised by an ethics committee of the University of Granada (2966/CEIH/2022). In this case, the participants gave their written informed consent and were able to withdraw from the research at their own discretion. They were also informed that anonymity would be ensured at all times and that the data would be processed for scientific purposes only.

### 2.4. Data Analysis

For the comparative analysis, the IBM SPSS 25.0 statistical programme (SPSS, IBM, SPSS Statistics, v.25.0 Chicago, IL, USA) was used. The variables were tested for normality and for the homogeneity of variance using the Kolmogorov–Smirnov test. In addition, a one-factor ANOVA was used, and the statistically significant differences were determined throughout using Pearson’s chi-squared test (*p* ≤ 0.05). Likewise, the magnitude of the difference in the effect size (ES) was obtained with Cohen’s standardised d-index [31], interpreted as null (≤0.19), small (0.20–0.49), medium (0.50–0.79) and large (≥0.80). Subsequently, we studied the normality of the sample using the Kolmogorov–Smirnov test.

**Figure 1:**
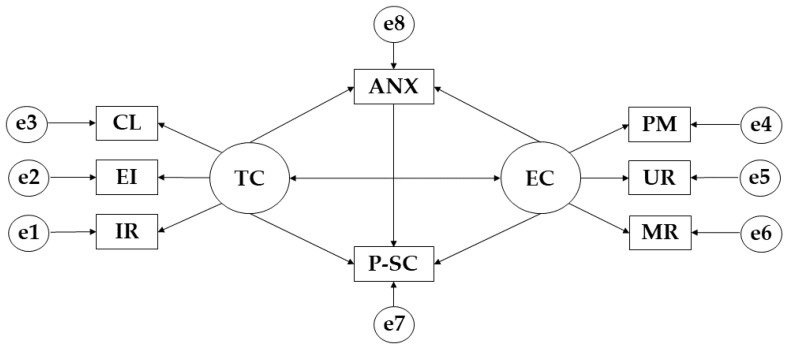
Proposed Theoretical Model. **Note:** Ego climate (EC); Punishment for mistakes (PM); Unequal recognition (UR); Member rivalry (MR); Physical self-concept (P-SC); Anxiety (ANX); Task climate (TC); Cooperative learning (CL); Effort/improvement (EI); Important role (IR).

For the development of the structural equation models, the IBM SPSS Amos 26.0 software (IBM Corp., Armonk, NY, USA) was used to establish the relationships between the variables that make up the theoretical model (Figure 1). In this case, a model was developed for each of the categories that make up the practice of physical activity. Each of the models is composed of two exogenous variables (TC; EC) and eight endogenous variables (IR; EI; CL; PM; UR; MR; ANX; P-SC). For these latter variables, a causal explanation was carried out on the basis of the observed associations between the measurement reliability and indicators, so that the measurement error was included in the model. In terms of the direction of the arrows, the unidirectional arrows represent the lines of influence between the latent variables, which are interpreted on the basis of the regression weights. Finally, with regard to the level of significance, two levels of 0.05 and 0.001 were established using Pearson’s chi-squared test.

Finally, the model was evaluated by estimating its parameters (Figure 1). Following the established criteria [32,33], the goodness of fit should be evaluated on the chi-squared test, whose values associated with *p* and are non-significant indicate a good fit of the model. For a good fit, the values of the comparative fit index (CFI), the goodness of fit index (GFI) and the incremental reliability index (IFI) should be greater than 0.900, while for the root mean square approximation (RMSEA), the values should be less than 0.100.

## 3. Results

### 3.1. Comparative Analysis

Table 1 shows the results obtained from the comparative analysis. Participants who practice more than 300 min of weekly physical activity show higher levels of physical self-concept than those who practice between 150 and 300 min (3.15 ± 0.68) and less than 150 min (2.55 ± 0.70). Subjects who practice less than 150 min of physical activity per week, present higher values of anxiety (0.97 ± 0.69) in comparison with those who practice between 150 and 300 min per week (0.77 ± 0.58) and with those who claim to practice more than 300 min (0.71 ± 0.61). Furthermore, observations were made on how, in cooperative learning, those participants who claim to practice less than 150 min of weekly physical exercise (3.80 ± 1.01), obtain lower scores compared to those who practice more than 300 min (4.14 ± 0.76). Continuing with effort improvement, it was observed that students who practice less than 150 min of physical activity per week (3.77 ± 0.84) have lower scores than those participants who practice between 150 and 300 min (3.98 ± 0.67) and more than 300 min per week (4.01 ± 0.66). Finally, focusing on the important role, it was observed that those participants who practice less than 150 min of physical activity (3.86 ± 0.92) have lower scores than those who claim to practice more than 300 min of physical exercise per week (4.15 ± 0.74).

### 3.2. Structural Equation Model Analysis

Regarding structural equation models, the model developed and proposed for participants with less than 150 min of weekly physical activity showed a good fit for all indices. The chi-squared analysis showed a non-significant *p*-value (X^2^ = 18.197; df = 16; pl = 0.312), but these data cannot be interpreted independently due to the influence of susceptibility and sample size [34], so other standardised fit indices were used. The comparative fit index (CFI), the normalised fit index (NFI), the incremental fit index (IFI) and the Tucker–Lewis index (TLI) all scored above 0.900. In addition, the root mean square error of the approximation analysis (RMSEA) obtained a value of 0.033.

**Figure 2:**
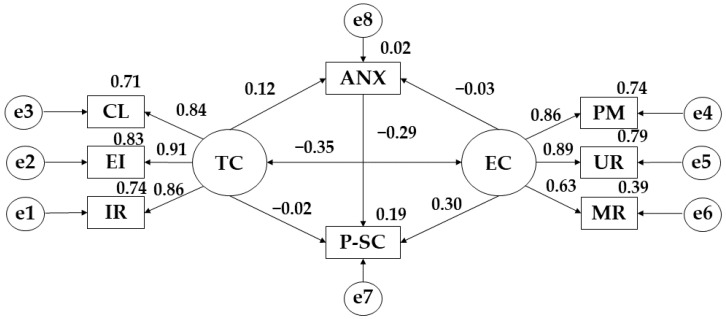
Theoretical model proposed for participants practising less than 150 min of PA per week. **Note:** Task climate (TC); Cooperative learning (CL); Effort/improvement (EI); Important role (IR); Ego climate (EC); Punishment for mistakes (PM); Unequal recognition (UR); Member rivalry (MR); Physical self-concept (P-SC); Anxiety (ANX).

Looking at the results obtained in Figure 2 and Table 2, it can be seen that there is a positive relationship between physical self-concept and task climate (r = 0.115) and a negative relationship with ego climate (r = −0.029). Continuing with the results, there are positive relationships between task climate and important role (r = 0.860), effort/improvement (*p* ≤ 0.001; r = 0.913) and cooperative learning (*p* ≤ 0.001; r = 0.840). For ego climate, positive relationships are also obtained between punishment for mistakes (r = 0.862), unequal recognition (*p* ≤ 0.001; r = 0.888) and rivalry between group members (*p* ≤ 0.001; r = 0.628). Focusing attention on the relationships observed for anxiety, a positive relationship is observed with ego climate (r = 0.298). In contrast, anxiety is negatively related to task climate (r = −0.025) and physical self-concept (r = −0.291). Finally, a negative relationship is observed between ego climate and task climate (r = −0.353).

Continuing with the model developed for participants who practice between 150 and 300 min of physical activity per week, the chi-squared analysis showed a significant value (X^2^ = 51.568; df = 16; pl = 0.000). The comparative fit index (CFI) analysis obtained a value of 0.944, which represents an excellent score. The normalised fit index (NFI) analysis obtained a value of 0.922, the incremental fit index (IFI) was 0.945 and the Tucker–Lewis index (TLI) obtained a value of 0.902, all of which were excellent. In addition, the root mean square error of approximation analysis (RMSEA) also obtained a value of 0.048.

**Figure 3:**
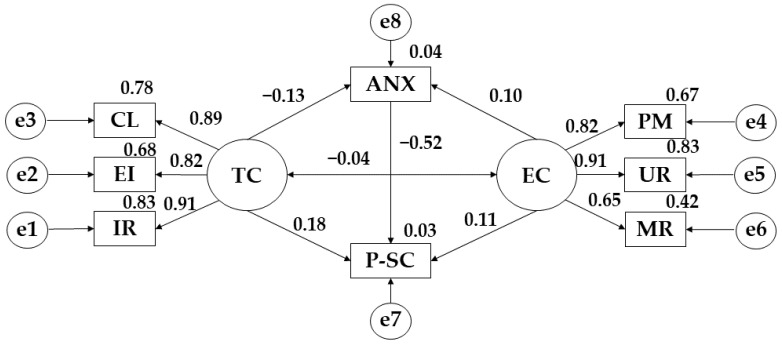
Theoretical model proposed for participants practising between 150 and 300 min of PA per week. **Note:** Task climate (TC); Cooperative learning (CL); Effort/improvement (EI); Important role (IR); Ego climate (EC); Punishment for mistakes (PM); Unequal recognition (UR); Member rivalry (MR); Physical self-concept (P-SC); Anxiety (ANX).

Figure 3 and Table 3 show that there is a positive relationship between the two motivational climates (TC; EC) and physical self-concept (r = 0.177) (r = 0.111). Likewise, a negative relationship is observed between anxiety and task climate (r = −0.130) On the contrary, a positive relationship between anxiety and ego-climate is observed (r = 0.099). Regarding task climate, a positive relationship is observed with important role (r = 0.912), effort/improvement (*p* ≤ 0.001; r = 0.823) and cooperative learning (*p* ≤ 0.001; r = 0.885). For ego climate, positive relationships are obtained with punishment for mistakes (r = 0.819), unequal recognition (*p* ≤ 0.001; r = 0.913) and rivalry between group members (*p* ≤ 0.001; r = 0.649). Continuing with the relationship between physical self-concept and anxiety, a negative relationship is evident (r = −0.036), occurring exactly the same between ego climate and task climate (*p* ≤ 0.001; r = −0.520).

The model developed for participants who practice more than 3 h of physical activity per week, the chi-squared analysis showed a significant value (X^2^ = 54.763; df = 16; pl = 0.000). The comparative fit index (CFI), the normalised fit index (NFI), the incremental fit index (IFI) and the Tucker–Lewis index (TLI) all scored above 0.900. The root mean square error of approximation analysis (RMSEA) obtained a value of 0.053.

**Figure 4:**
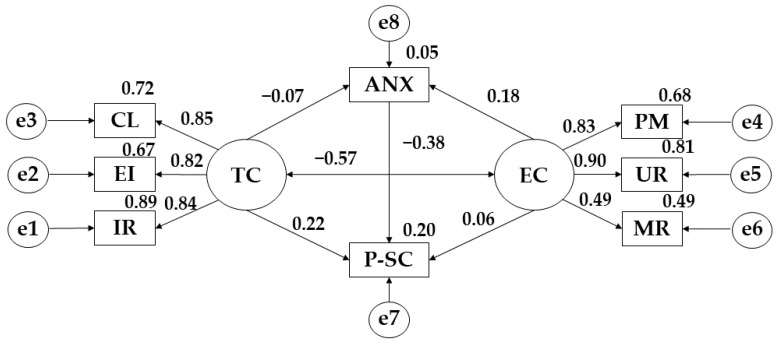
Theoretical model proposed for participants practising more than 300 min of PA per week. **Note:** Task climate (TC); Cooperative learning (CL); Effort/improvement (EI); Important role (IR); Ego climate (EC); Punishment for mistakes (PM); Unequal recognition (UR); Member rivalry (MR); Physical self-concept (P-SC); Anxiety (ANX).

In Figure 4 and Table 4, a negative relationship was obtained between task climate and anxiety (r = −0.068). On the contrary it observed a positive relationship with anxiety and ego climate (r = 0.184). Continuing with this last variable, a positive relationship was observed with punishment for errors (r = 0.826), unequal recognition (*p* ≤ 0.001; r = 0.889) and rivalry between group members (*p* ≤ 0.001; r = 0.493). Looking at task climate, it is positively related to important role (r = 0.941), effort/improvement (*p* ≤ 0.001; r = 0.817) and cooperative learning (*p* ≤ 0.001; r = 0.846). Focusing attention on physical self-concept, it shows a positive relationship with ego climate (r = 0.064) and task climate (r = 0.223). However, a negative relationship is observed with anxiety (r = −0.378). Finally, ego climate has a negative relationship with task climate (*p* ≤ 0.001; r = −0.571).

## 4. Discussion

The present study shows the relationships between the motivational climate developed towards sports practice, anxiety, physical self-concept and weekly physical activity practice time. In this way, the results obtained respond to the initially stated objectives, and therefore, the present discussion aims to compare the results obtained with those already obtained in other research.

Based on the results, it can be seen that those participants who do not meet the minimum weekly physical activity requirements have lower physical self-concept scores than those who exceed the recommended limit. Very similar results were obtained by Duarte-Climents et al. [35] where Bilgin et al. [36] state that the practice of physical activity helps to improve the subjects’ fitness and, therefore, their physical self-concept and mental self-image. Furthermore, Callow et al. [37] argue that improving physical fitness and body image brings benefits to both the physical and mental health of individuals.

Continuing with anxiety, it is observed that those subjects who do not meet the minimum weekly physical activity requirements show higher levels of anxiety than those who exceed the recommended limit. Very similar results were concluded by Kayani et al. [16] where Ubago-Jiménez et al. [38] state that the practice of physical activity helps to channel disruptive states such as anxiety and stress, this is due to the segregation of neurotransmitters released during physical sports practice, such as serotonin and dopamine.

Focusing attention on the variables that make up the task climate, it is observed that the greater the weekly physical activity practice, the higher the scores obtained in each of these variables. Very similar results were obtained by Kuipers et al. [39], who state that when the practice of physical activity is oriented towards intrinsic motivations, more efficient learning and the development of attitudes that foster cooperation and companionship are achieved. However, for the ego climate, it is observed that those participants who comply with the recommendations obtain higher scores than those who exceed the limit. Jenkins et al. [40] argue that when physical activity is oriented towards ego climate, extrinsic values, such as competition, become more important. Furthermore, Sokic et al. [41] state that training times at the professional level are oriented towards the highest possible performance.

Continuing with the structural equation models developed, focusing attention on the model proposed for subjects who practice less than 150 min of weekly physical activity, it can be seen how the ego climate has a negative impact on the development of physical self-concept. Very similar results were obtained by Babic et al. [42] where they state that when the minimum standards of weekly physical activity practice are not met, and they begin to practice any type of sport and a poor conception of physical fitness is produced. Likewise, a positive relationship is also observed between anxiety and ego climate, where Ruffault et al. [13] state that when the practice of physical activity begins to be carried out in a professional manner, anxiety is fostered, due to the fact that, at a professional level, sport demands a greater degree of pressure to achieve victory. Considering the relationship between anxiety and physical self-concept, a negative relationship is observed, with Cueli et al. [43] stating that anxiety has a negative impact on the development of self-concept, as it distorts the mental image that subjects have of themselves.

Continuing with the model developed for subjects who practice between 150 and 300 min of activity per week, a negative relationship between task climate and anxiety is observed. Very different results were obtained by Zhang et al. [44]. However Chu et al. [45] argue that when the practice of physical exercise is oriented towards competition, there is a negative relationship with the ideas that the task climate conveys. Likewise, a positive relationship is also obtained between ego climate and task climate with physical self-concept. These results are very similar to those obtained by Liu et al. [46] who stated that the practice of physical activity reports improvements in the area of mental health, in this case, in the physical self-concept.

Focusing on the model developed for participants who practice more than 300 min of physical activity per week, a positive relationship between ego climate and anxiety is evident, with Petzold et al. [47] stating that when the practice of physical activity is oriented towards extrinsic motivations, anxiety increases. Likewise, there is also evidence of a negative relationship between physical self-concept and anxiety, with Fantineli et al. [48] stating that an adequate body image and acceptance of the physical state of each subject brings benefits in the mental area of the subjects.

Finally, this research highlights the importance of the motivational climate oriented towards the practice of physical sports. In this case, the study carried out by Girard and Lemoyne [49] highlights the relevance of the physical education teacher in developing favourable behaviours towards sports practice. This will have a positive impact on the health of young people. Likewise, the study by Laxdal et al. [50] affirms that when physical education classes are oriented towards the homework climate, a positive attitude towards the practice of physical activity is developed in future stages, in such a way that healthy behaviours are incorporated into the lifestyle of young people.

## 5. Limitations and Future Perspectives

This research, despite meeting the objectives set out, has a series of limitations. The first of these is that, as this is a cross-sectional study, it only allows us to establish causal relationships at that point in time. Likewise, the sample belongs to a very specific geographical area, which means that generalisations cannot be made to a wider area of the national or regional geography. Furthermore, the instruments used, despite having been validated by the scientific community, have an intrinsic error in their measurement. Likewise, the practice of physical activity has been measured over time, leaving aside its intensity. The sample is homogeneous, with more than half of the population being female.

It should be noted that, as a strength, the research presents a multi-group structural equation analysis which allows us to observe in greater detail the differences present in the three study groups. Another point to note is that the data are fully reliable, as instruments and data analysis accepted by the scientific community have been used.

With a view to future perspectives, the present study highlights the different benefits of the practice of physical sports on a psychological level. One function of the present research is that of a pilot study for the elaboration of a longitudinal study that will focus on the motivation developed towards physical exercise and the different benefits it brings to peoples’ physical and mental health.

## 6. Conclusions

The present research shows the relationships between the motivational climate developed from the practice of physical activity and its relationship with anxiety and physical self-concept in trainee physical education teachers.

Based on the comparative analysis, it is observed that participants who exceed the weekly physical activity limits obtain better results in physical self-concept, anxiety, cooperative learning, effort/improvement and important role. It is also observed that those who comply with the amount of time established by the WHO show better results in punishment for errors, unequal recognition and rivalry between group members.

Focusing attention on the model developed for participants who practice less than 150 min of activity per week, negative relationships are observed between ego climate,-and physical self-concept, anxiety and task climate, anxiety and physical self-concept and between ego climate and task climate. Moreover, positive relationships are shown between task climate and physical self-concept and anxiety and ego climate.

Continuing with the model proposed for participants who practice between 150 and 300 min, negative relationships are obtained between anxiety and task climate, anxiety and physical self-concept and ego climate and task climate. However, positive relationships are shown between anxiety and ego climate, physical self-concept and task climate and physical self-concept and ego climate.

The model developed for participants who practice more than 300 min of physical activity per week, positive relationships are observed between anxiety and ego climate, physical self-concept and task climate and ego climate and physical self-concept. However, negative relationships are obtained for anxiety and task climate, anxiety and physical self-concept and ego climate and task climate.

Finally, a practical application lies in the subject of physical education in early childhood development. In this case, the physical education teacher is of great importance in the development of a certain motivational climate, because depending on how he/she guides his/her classes, he/she can develop a positive or negative attitude towards the pupils. The development of a negative attitude can be detrimental to young people in later stages of physical and mental development.

## Figures and Tables

**Table 1:** Comparative analysis of the variables.

		N	M	SD	F	*p*	ES (d)	95%CI
**P-SC**	**Less than 150 min**	128	2.55	0.70	51.074	≤0.05 ^a,b^	0.871 ^a^ 1.067 ^b^	[0.631; 1.111] ^a^ [0.842; 1.292] ^b^
	**Between 150 and 300 min**	170	3.15	0.68				
	**More than 300 min**	270	3.37	0.80				
**ANX**	**Less than 150 min**	128	0.97	0.69	7.688	≤0.05 ^a,b^	0.310 ^a^ 0.408 ^b^	[0.079; 0.541] ^a^ [0.194; 0.621] ^b^
	**Between 150 and 300 min**	170	0.77	0.58				
	**More than 300 min**	270	0.71	0.61				
**CL**	**Less than 150 min**	128	3.80	1.01	6.509	≤0.05 ^b^	0.400 ^b^	[0.186; 0.613] ^b^
	**Between 150 and 300 min**	170	4.00	0.87				
	**More than 300 min**	270	4.14	0.76				
**EI**	**Less than 150 min**	128	3.77	0.84	5.011	≤0.05 ^b,c^	0.323 ^c^ 0.331 ^b^	[0.092; 0.554] ^c^ [0.118; 0.544] ^b^
	**Between 150 and 300 min**	170	3.98	0.67				
	**More than 300 min**	270	4.01	0.66				
**IR**	**Less than 150 min**	128	3.86	0.92	5.280	≤0.05 ^b^	0.362 ^b^	[0.149; 0.575] ^b^
	**Between 150 and 300 min**	170	4.03	0.89				
	**More than 300 min**	270	4.15	0.74				
**PM**	**Less than 150 min**	128	2.32	0.81	0.779	0.460	NP	NP
	**Between 150 and 300 min**	170	2.44	0.82				
	**More than 300 min**	270	2.38	0.77				
**UR**	**Less than 150 min**	128	2.72	1.04	0.487	0.615	NP	NP
	**Between 150 and 300 min**	170	2.72	1.02				
	**More than 300 min**	270	2.64	1.01				
**MR**	**Less than 150 min**	128	2.63	0.89	1.029	0.358	NP	NP
	**Between 150 and 300 min**	170	2.78	0.88				
	**More than 300 min**	270	2.73	0.93				

**Note 1:** ^a^ Differences between physical practice of less than 150 min and between 150 and 300 min. ^b^ Differences between physical practice of less than 150 min and of more than 300 min. ^c^ Differences existing between less than 150 min with more than 300 min. **Note 2:** Ego climate (EC); Punishment for mistakes (PM); Unequal recognition (UR); Member rivalry (MR); Physical self-concept (P-SC); Task climate (TC); Cooperative learning (CL); Effort/improvement (EI); Important role (IR); Anxiety (ANX).

**Table 2:** Structural model for participants practising for less than 150 min.

Associations between Variables	R.W.				S.R.W.
	Estimations	S.E.	C.R.	*p*	Estimations
P-SC ← TC	0.103	0.090	1.150	0.250	0.115
P-SC ← EC	−0.029	0.102	−0.281	0.779	−0.029
IR ← TC	1.000				0.860
EI ← TC	0.968	0.075	12.874	***	0.913
CL ← TC	1.073	0.091	11.774	***	0.840
PM ← EC	1.000				0.862
UR ← EC	1.315	0.133	9.873	***	0.888
MR ← EC	0.789	0.107	7.382	***	0.628
ANX ← TC	−0.022	0.081	−0.270	0.787	−0.025
ANX ← EC	0.295	0.093	3.156	**	0.298
ANX ← P-SC	−0.286	0.080	−3.593	***	−0.291
EC ← → TC	−0.197	0.060	3.285	**	−0.353

**Note 1:** regression weights (R.W.); standardised regression weights (S.R.W.); estimation error (S.E.); critical ratio (C.R.). **Note 2:** ego climate (EC); punishment for mistakes (PM); unequal recognition (UR); member rivalry (MR); physical self-concept (P-SC); anxiety (ANX); task climate (TC); cooperative learning (CL); effort/improvement (EI); important role (IR). **Note 3:** ** *p* ≤ 0.05; *** *p* ≤ 0.001.

**Table 3:** Structural model for participants practising between 150 and 300 min of PA per week.

Associations between Variables	R.W.				S.R.W.
	Estimations	S.E.	C.R.	*p*	Estimations
ANX ← TC	−0.092	0.068	−1.353	0.176	−0.130
ANX ← EC	0.085	0.083	1.023	0.306	0.099
IR ← TC	1.000				0.912
EI ← TC	0.685	0.048	14.196	***	0.823
CL ← TC	0.963	0.060	15.929	***	0.885
PM ← EC	1.000				0.819
UR ← EC	1372	0.121	11.388	***	0.913
MR ← EC	0.861	0.098	8.798	***	0.649
P-SC ← TC	0.157	0.086	1.823	0.068	0.177
P-SC ← EC	0.117	0.104	1.124	0.261	0.111
P-SC ← ANX	−0.044	0.096	−0.458	0.647	−0.036
EC ← → TC	−0.273	0.053	−5.173	***	−0.520

**Note 1:** regression weights (R.W.); standardised regression weights (S.R.W.); estimation error (S.E.); critical ratio (C.R.). **Note 2:** ego climate (EC); punishment for mistakes (PM); unequal recognition (UR); member rivalry (MR); physical self-concept (P-SC); anxiety (ANX); task climate (TC); cooperative learning (CL); effort/improvement (EI); important role (IR). **Note 3:** *** *p* ≤ 0.001.

**Table 4:** Structural model for participants practising more than 300 min.

Associations between Variables	R.W.				S.R.W.
	Estimations	S.E.	C.R.	*p*	Estimations
ANX ← TC	−0.060	0.071	−0.844	0.399	−0.068
ANX ← EC	0.179	0.081	2.206	**	0.184
IR ← TC	1.000				0.941
EI ← TC	0.774	0.044	17.704	***	0.817
CL ← TC	0.920	0.049	18.842	***	0.846
PM ← EC	1.000				0.826
UR ← EC	1.435	0.112	12.798	***	0.899
MR ← EC	0.725	0.093	7.805	***	0.493
P-SC ← ANX	−0.491	0.075	−6.584	***	−0.378
P-SC ← TC	0.255	0.085	2.991	**	0.223
P-SC ← EC	0.081	0.098	0.828	0.407	0.064
EC ← → TC	−0.254	0.037	−6.820	***	−0.571

**Note 1:** Regression weights (R.W.); Standardised regression weights (S.R.W.); Estimation error (S.E.); Critical ratio (C.R.). **Note 2:** Ego climate (EC); Punishment for mistakes (PM); Unequal recognition (UR); Member rivalry (MR); Physical self-concept (P-SC); Task climate (TC); Cooperative learning (CL); Effort/improvement (EI); Important role (IR); Anxiety (ANX). **Note 3:** ** *p* ≤ 0.05; *** *p* ≤ 0.001.

## Data Availability

The data used to support the findings of the current study are available from the corresponding author upon request.
